# RCN4GSC Workshop Report: Managing Data at the Interface of Biodiversity and (Meta)Genomics, March 2011

**DOI:** 10.4056/sigs.3156511

**Published:** 2012-07-28

**Authors:** Robert J. Robbins, Linda Amaral-Zettler, Holly Bik, Stan Blum, James Edwards, Dawn Field, George Garrity, Jack A. Gilbert, Renzo Kottmann, Leonard Krishtalka, Hilmar Lapp, Carolyn Lawrence, Norman Morrison, Éamonn Ó Tuama, Cynthia Parr, Inigo San Gil, David Schindel, Lynn Schriml, David Vieglas, John Wooley

**Affiliations:** 1 University of California San Diego, La Jolla, California, USA; 2 Josephine Bay Paul Center for Comparative Molecular Biology and Evolution, Marine Biological Lab, Woods Hole, Massachusetts, USA; 3 Hubbard Center for Genome Studies, University of New Hampshire, Durham, NH USA; 4 Center for Applied Biodiversity Informatics, California Academy of Sciences, San Francisco, California, USA; 5 Encyclopedia of Life, Smithsonian Institution, Washington, DC, USA; 6 Centre for Ecology & Hydrology, Maclean Building, Crowmarsh Gifford, Wallingford, Oxfordshire, United Kingdom; 7 Department of Microbiology and Molecular Genetics, Michigan State University, East Lansing, Michigan, USA; 8 Argonne National Laboratory, 9700 South Cass Avenue, Argonne, IL, USA.; 9 Department of Ecology and Evolution, University of Chicago, 5640 South Ellis Avenue, Chicago, IL, USA; 10 Microbial Genomics Group, Max Planck Institute for Marine Microbiology, Bremen, Germany; 11 University of Kansas Natural History Museum, Lawrence, KS, USA; 12 National Evolutionary Synthesis Center (NESCent), Durham, North Carolina, USA; 13 USDA-ARS Corn Insects and Crop Genetics Research Unit and Department of Genetics, Development, and Cell Biology, 1034 Crop Genome Informatics Laboratory, Iowa State University, Ames, Iowa, USA; 14 School of Computer Science, Kilburn Building, University of Manchester, Oxford Road, Manchester, England UK M13 9PL; 15 Global Biodiversity Information Facility, GBIF Secretariat, Copenhagen, Denmark; 16 Department of Biology, LTER Network Office, University of New Mexico, Albuquerque, NM USA; 17 Consortium for the Barcode of Life, National Museum of Natural History, Smithsonian Institution, Washington, DC 20013-7012 USA; 18 Institute for Genome Sciences, University of Maryland School of Medicine, Baltimore, MD 20742 USA

## Abstract

Building on the planning efforts of the RCN4GSC project, a workshop was convened in San Diego to bring together experts from genomics and metagenomics, biodiversity, ecology, and bioinformatics with the charge to identify potential for positive interactions and progress, especially building on successes at establishing data standards by the GSC and by the biodiversity and ecological communities. Until recently, the contribution of microbial life to the biomass and biodiversity of the biosphere was largely overlooked (because it was resistant to systematic study). Now, emerging genomic and metagenomic tools are making investigation possible. Initial research findings suggest that major advances are in the offing. Although different research communities share some overlapping concepts and traditions, they differ significantly in sampling approaches, vocabularies and workflows. Likewise, their definitions of ‘fitness for use’ for data differ significantly, as this concept stems from the specific research questions of most importance in the different fields. Nevertheless, there is little doubt that there is much to be gained from greater coordination and integration. As a first step toward interoperability of the information systems used by the different communities, participants agreed to conduct a case study on two of the leading data standards from the two formerly disparate fields: (a) GSC’s standard checklists for genomics and metagenomics and (b) TDWG’s Darwin Core standard, used primarily in taxonomy and systematic biology.

## Background

The Genomic Standards Consortium (GSC) is an international working body with the mission of working towards richer descriptions of genomic and metagenomic data through the development of standards and tools for supporting the consistent documentation of contextual information (source, preparation, etc.) about sequences. Established in September 2005, the community includes representatives from the International Nucleotide Sequence Database Collaboration (INSDC), major genome sequencing centers, bioinformatics groups, and a range of research institutions.

In 2009, the National Science Foundation funded a Research Coordination Network (RCN) project for the GSC (RCN4GSC, hosted at UCSD, with John Wooley as PI) to continue the GSC’s work of promoting and integrating standards for recording contextual information about the sample, nucleic acid processing and analysis associated with genomic and metagenomic data [1].

In general, NSF RCN awards are intended to advance a field or create new directions by supporting the coordination of research, training and educational activities across disciplinary, organizational, geographic and international boundaries, with the development of community standards for data and meta-data being especially encouraged.

The RCN4GSC project has the specific goal of extending prior GSC work on checklists to assist in the harmonization of existing ecological data standards [2], such as Ecological Metadata Language (EML, maintained by the Knowledge Network for Biocomplexity — KNB) [3] and biodiversity standards such as Darwin Core (DwC, maintained by the Taxonomic Databases Working Group — TDWG) [4], and also to engage environmental research programs such as the Global Lake Ecological Observatory Network (GLEON), the National Ecological Observatory Network (NEON), and Long Term Ecological Research (LTER).^1^

At the 9^th^ GSC meeting (GSC9, 28-30 April 2010) [5], a session was dedicated to considering linkages between the GSC and the biodiversity community. A Biodiversity Working Group (BDWG) was formed to explore the intersection between the GSC and communities working at the forefront of biodiversity research [6]. The BDWG is an open organization, with membership available to anyone interested in assisting in its work.^2^ BDWG is chaired by Norman Morrison (University of Manchester).

**The GSC has been instrumental in establishing and promulgating a series of minimum checklist standards for genomic data within the, Minimum Information about Any (x) Sequence (MIxS) framework [7]:**
- minimum information about a genome sequence — MIGS [6];
- minimum information about a metagenome sequence — MIMS [6]; and
- minimum information about a marker gene sequence — MIMARKS [7] (including the extension to environmental packages to better describe environmental conditions.
  The utility of molecular methods in studying biodiversity has been recognized for some time and this joint area is receiving increasing attention from a variety of groups. For example, in January 2011, the National Evolutionary Synthesis Center (NESCent) hosted a catalysis meeting entitled “high-throughput biodiversity research using eukaryotic metagenetics” to discuss the multitude of informatics challenges associated with this new era of biodiversity research, ultimately producing a number of recommendations, including:
- The collection of high-throughput data must be designed to have maximum global usefulness (the coordinated use of common genetic loci), and be transferrable as sequencing technology evolves and the number of potential target loci expands.
- Databases and cyber resources must meet the needs of the scientific community; at present, eukaryote-focused resources are lacking, but rapid progress can be made by leveraging tools and resources from the microbial community.
- The effective use of high-throughput methods presently requires specialist knowledge and substantial computational skills — in order to engage a wider audience of non-computationally trained biologists and ecologists, there is a pressing need for intuitive metadata terminology and analytical pipelines (e.g. graphical interfaces).

In keeping with the coordination and collaboration goals of RCN4GSC, contact was made with the organizers of the NESCent meeting to initiate linkages and two participants in the NESCent meeting (including one of the organizers) also participated in this present RCN4GSC workshop.

Genomic methods will have an increasingly important role to play in biodiversity, ecological and conservation research, where data standards such as Darwin Core (DwC) and Ecological Metadata Language (EML) have already been developed. Recognizing that effective data management across biodiversity and (meta)genomics will require the joint use of shared standards, the GSC convened this planning meeting to begin exploring opportunities and challenges associated with data management at the interface of biodiversity and (meta)genomics (≡ both genomics and metagenomics).

## Purposes of the Meeting

Because work at this interface is expanding rapidly, efforts to facilitate appropriate data management must also occur rapidly. Therefore this meeting was convened (with some urgency) as a planning session, aimed at getting as much information “on the table” as possible. Specifically, the goal was to identify potential for positive interactions and progress, especially building on successes at establishing data standards by the GSC and by the biodiversity and ecological communities.

**The purposes of the workshop were**
- To identify and characterize opportunities, challenges, and benefits that occur when genomic and metagenomic technologies, methods, and standards (for data exchange and contextual data and metadata) are brought to bear upon studies of biodiversity (the interface),
- To identify and characterize the methods and tools necessary to deliver benefits and to address the challenges identified above,
- To assess the adequacy of current technology and infrastructure in this context, and to identify gaps and inadequacies in current capabilities, methods, approaches, or standards,
- To propose steps to remediate identified deficiencies and advance the interface,
- To provide input for a white paper, ultimately to be published in *Standards in Genomic Sciences* (*SIGS* — the GSC’s e-journal) documenting key aspects of the interface,
- To identify key participants who should be added (besides extant GSC and GSC Biodiversity Working Group members) to contribute, edit, and critique the white paper through email, teleconference, small working groups or other vehicles. (The white paper will be discussed at the international GSC meetings, GSC 11 and 12, over the course of 2011, extensively reviewed by GSC and its Working Group, and all of the attendees of this March workshop.)
- To establish a preliminary outline of what topics would need to be addressed at a large scale “GSC-Biodiversity-Interface” meeting (should one occur), and
- To identify and validate what organizations and individuals would be essential for the large scale interface meeting (or if this is too complex, what sorts of more modest scale interactions would be necessary to establish an effective set of networks for the GSC among diverse subfields).

## Participants

In keeping with our sense of urgency, the goal was to convene a meeting quickly to *initiate* activities in this area. Therefore, attendees were invited as individuals — not as representatives of an organization or institution or another. While this allowed us to be nimble in initiating the process, we recognize the importance of ultimately achieving general community and institutional consensus before the adoption of final recommendations regarding standards can occur.

At the same time, efforts were made to be “representative or inclusive enough” on a scale that should allow actual planning to be done and to provide appropriate “future-proofing” of the implemented ideals.

## Activities and Analysis

The attendees discussed both opportunities and challenges associated with the interface of traditional biodiversity surveys and (meta)genomic analysis of biodiversity. Recognizing that work at the interface could revolutionize our understanding of biology, the group spent time laying out both a future vision for integrated data management and an assessment of initial steps that offer the greatest opportunity for immediate pay back.

## Conclusions

Participants at the planning meeting unanimously concluded that the application of genomic and metagenomic tools to studies of biodiversity and ecology are sure to deepen our understanding of those fields. Expanding the range of species subjected to study by (meta)genomic tools beyond prokaryotes and ‘model’ eukaryotes would broaden our understanding of those species. This greater depth and breadth could transform our understanding of all of biology.

Until recently, the contribution of microbial life to the biomass and biodiversity of the biosphere was largely overlooked (because it was resistant to systematic study). Now, emerging genomic and metagenomic tools are making investigation possible. Initial research findings suggest that major advances are in the offing.

Although different research communities share some overlapping concepts and traditions, they differ significantly in sampling approaches, vocabularies and workflows. Likewise, their definitions of ‘fitness for use’ for data differ significantly, as this concept stems from the specific research questions of most importance in the different fields. Nevertheless, there is little doubt that there is much to be gained from greater coordination and integration.

**For instance:**
- Study samples, software, laboratory capabilities and capacity, the database contents, and supporting informatics infrastructure of each field may be highly useful to the other. If the fields can agree to openly share these resources, each can leverage benefits based on economies of scale and avoid unnecessarily duplicative expenditures.
- Building a shared understanding of the structures of information across these fields is critical to a fuller comprehension of what drives and limits biological diversification over space and time. It is only by bringing together and trying to integrate explanations across dimensions of biodiversity that we can build robust, testable models of how nature works.

**Looking ahead, meeting attendees outlined a vision of how both biodiversity and genomics data sets might be jointly expanded:**
- Extending traditional biodiversity data by adding specimen sequence data to the data about the specimen.
- Extending traditional biodiversity data by augmenting specimen data with metagenomic data taken from associated microbiomes (gut, surface, various cavities and orifices, root nodules, etc) of the specimen.
- Extending traditional biodiversity data by adding metagenomic data taken from the surrounding environment (soil, water, air) to the voucher descriptions of the environment from which the specimen was collected (particularly important for plants and sessile animals).
- Extending metagenomic data by adding a full collections-oriented (e.g., Darwin Core) description of the host from which a commensal microbial metagenomics sample was collected. For example, instead of merely noting that a metagenomics sample was taken from the gut of a particular species of beetle, record also sufficient information about the individual beetle that it could be accessioned as a voucher or type specimen into a good entomological collection.
- Extending environmental metagenomics data to include documentation of historical data about the ecosystem (both gross and micro-habitat) from which the sample was collected.
- Extending geospatial / environmental data to include metagenomic biodiversity data at a temporal level to enable modeling related to particular events.
- Extending genomic data by adding a full collections-oriented (e.g., Darwin Core) description of the individual from which the DNA was taken.
- Integrating all of the above with field ecology data systems, including GIS, so that geospatial queries could be made that range across genomic, organismal, taxonomic, ecological, environmental, and temporal variables.

Before these longer term goals can be achieved, initial steps must be taken to analyze the compatibility and complementarity of existing data standards. Therefore, the planning meeting attendees unanimously recommend that immediate efforts be initiated to compare and analyze the checklists of Darwin Core and GSC (the various MIxS checklists), develop a merged checklist approach, identify and develop test data sets to exercise such a merged approach, and design use cases that serve as showcase of these value added data sets. Specific recommendations follow.

## Recommendations

As a first step toward interoperability of the information systems used by the different communities, participants agreed to conduct a case study on two of the leading data standards from the two formerly disparate fields: (a) GSC’s standard checklists for genomics and metagenomics and (b) TDWG’s Darwin Core standard, used primarily in taxonomy and systematic biology.

**The case study would involve:**
- Comparing the checklists of the two standards, looking for synonymies as well as conceptual gaps
- Promoting georeferencing and designation of voucher specimens as universal standards in biodiversity research
- Promoting the development of use cases that would help to define fitness for use and the data that would be required across standards
- Testing the applicability of each community’s existing software tools on the other’s databases
- Promoting the development of new tools that work across all biodiversity databases, especially for error detection and correction
- Establishing interdisciplinary knowledge-exchange networks with interactive, open and very broad participation as a mechanism (sometimes called *crowdsourcing*) to monitor and improve data quality and completeness
- Seeking interoperability, economies of scale and mutual intellectual benefits through common data standards, subscribed to by these and other communities of practice (e.g., ecoinformatics, physiology).

## Timeline for 2011

Efforts by the BDWG to facilitate the development of useful data standards and procedures for the interface of biodiversity with genomics and metagenomics will be an ongoing activity. Here (and in subsequent BDWG reports) we provide a timeline of events. Italics indicate that the suggested activity has already occurred; plain text that the activity is proposed.

Mar: *Convene a BDWG planning meeting to initiate an analysis of biodiversity, genomics, and meta-genomics: opportunities and challenges.*

Apr: Introduce the BDWG biodiversity-interface initiative at GSC11 meeting, UK; invite the development of use cases.

May: Form an RCN Working Group with GSC and Darwin Core specialists.

Jul: Engage with DNA barcode standard through Consortium for the Barcode of Life working group.

Sep: Report and discuss progress on initiative at GSC12 meeting, Bremen, Germany.

Oct: Engage GBIF and EOL before and during TDWG meeting, 16-21 October, in New Orleans, Louisiana, US.

Nov: Discuss metadata capture, ecological sampling and analysis, NEON workshop, Boulder, CO.

Dec: Present and discuss biodiversity-interface initiative at Fourth International Barcode of Life Conference, Adelaide, Australia.

## Notes

^1^ Standards and tools are critical for extending capacity and capabilities into new fields. For example, the rising concern over marine genomic resources in areas beyond national jurisdiction is an area where standards become especially important for making data available and useable to a broad audience.

^2^ For further information, including contact details and upcoming GBWG events please visit: http://gensc.org/gc_wiki/index.php/Biodiversity_Working_Group
